# Gastro-ileal anastomosis bypass – Exploring for an expanding surgical treatment for diabetes in patients with low body mass index: Case series

**DOI:** 10.1016/j.ijscr.2020.01.051

**Published:** 2020-02-06

**Authors:** Rey Jesús Romero, Raquel Colorado-Subizar

**Affiliations:** Department of Bariatric and Metabolic Surgery, Bariatric and Metabolic Surgical Center Obesity Health, Marigalante 60 D02 Fracc. Las Américas, Boca del Río, Veracruz, ZC, 94299, México

**Keywords:** Metabolic surgery, Body mass index less 35, Diabetes surgery, Case report, Gastroileal, Anastomosis bypass, Low body mass index

## Abstract

**Introduction:**

Several metabolic operations have been created in an attempt to enhance the equilibrium between safety, efficacy and costs of accessible metabolic surgery in diabetic patients with low body mass index (BMI). The purpose of this study is to present the preliminary outcomes of a novel procedure.

**Methods:**

A Gastro-Ileal Anastomosis Bypass (GIA-B) was performed in 4 diabetic patients at Boca del Rio Hospital, Veracruz, México. The study was performed between March 2018 and October 2019. GIA-B was created at point to 300 cm from ileocecal valve that was held together with gastric antrum. Outcomes are presented and discussed. At average 14.7 months follow-up all the patients improved glycated hemoglobin(A1C), decrease antidiabetic medications and lost mild weight. Two patients had complete remission of type-2 diabetes mellitus. There were no postoperative complications.

**Results:**

GIA-B, have a considerable metabolic effect reaching improvement of the homeostatic parameters, specially A1C, in all the cases evaluated. GIA-B appears to be technically simple and the cost is considerably lower than other metabolic procedures, especially for the saving cartridges.

**Conclusions:**

GIA-B could be an alternative metabolic surgery for low-BMI diabetic patients, further studies are needed to explore this procedure.

## Highlights

• Metabolic surgery for patients with low body mass index has proven good outcomes.
• Novel metabolic procedures require a balance among effectivity, simplicity and cost.
• Gastro-Ileal anastomosis bypass is a simplification of Sigle Anastomosis Ileal Bypass.
• Gastro-ileal anastomosis bypass (GIA-B) improves glycated hemoglobin.

## 1 Introduction

Type-2 Diabetes Mellitus (T2D) is a significant public health issue. Bariatric and Metabolic surgery have been growing as an alternative treatment for obesity and T2D. In spite of the evidence, about 15 million people in the U.S. have morbid obesity but only 1 % of the clinically eligible population is treated through bariatric surgery [1], in diabetic eligible population for metabolic surgery tendency is probably similar, being the economic factor the most important cause.

In an effort to improve balance between advantages, safety and price, several new techniques have emerged as alternatives for metabolic surgery. Mahdy et al. [2] published a new concept in metabolic surgery, the Single Anastomosis Sleeve Ileal Bypass (SASI-B) with promising results. We took the concept of this operation, made technical changes and created, in our knowledge, a new concept, Gastro-Ileal Bypass (GIA-B). The purpose of this study is to present our preliminary results. This work has been reported in line with the the PROCESS guidelines [3].

## 2 Methods

Four patients were included in this prospective, single-center study prior the authorization of the ethical committee of the Boca del Rio General Hospital at Veracruz (community practice center), Mexico registered with number 018/2016. UIN register 5220. The study was performed between March 2018 and October 2019. Human studies were performed in accordance with the 1964 Helsinki Declaration and its later amendments or comparable ethical standards. One surgeon, with previous experience in bariatric/metabolic surgery, performed the procedures. The patients were informed in detail about the investigational nature of the procedure and signed the relevant consent form prior to their inclusion in the study. Inclusion criteria were [a] T2D with ≤15 years since diagnosis [b]; body mass index (BMI) between 25–32 kg/m^2^ [c]; Age 30–55 years [d] Uncontrolled T2D with glycated hemoglobin (A1C) ≥6. Every patient was previously assessed by specialists in internal medicine, nutrition and psychology; and pertinent preoperative studies were required. %EWL was calculated with the ideal body weight as that equivalent to a BMI of 25 kg/m^2^. We defined T2D remission according to the American Diabetes Association [4] as following: Partial remission included patients with hyperglycemia below diagnostic thresholds for diabetes, at least 1 year´s duration and without active pharmacologic therapy or ongoing procedures; and complete remission included patients with normal glycemic measures, at least 1 year´s duration and without active pharmacologic therapy or ongoing procedures. Surveillance were achieved by the multidisciplinary team, including specialist in surgery, nutrition, psychology, internal medicine and medical coordination. In this study, there was not loss to follow-up.

### 2.1 Technique gastro ileal anastomosis bypass (GIA-B)

**Fig. 1:**
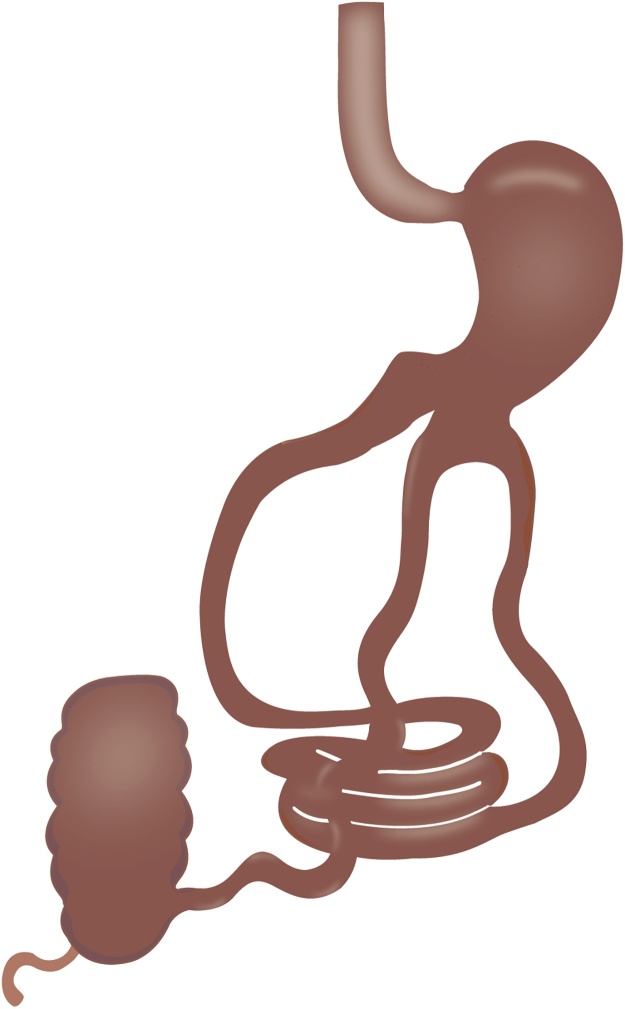
Gastro-Ileal Anastomosis Bypass (GIA-B). Anastomosis between ileum (to 300 cm from ileo-cecal valve) and stomach (antrum).

**Fig. 2:**
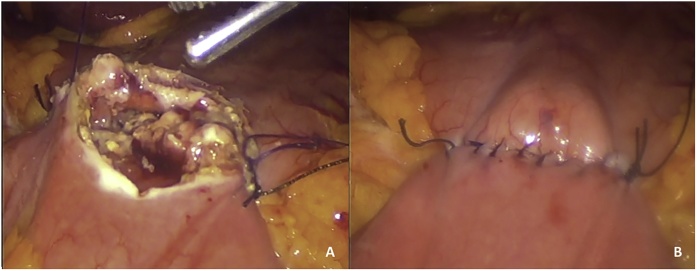
It shows creation of the laparoscopic manual anastomosis (A) between ileum and stomach and the anastomosis completed (B).

Under general anesthesia 4 trocars were place. A point from 300 cm from Ileocecal valve was identified and held together with gastric antrum, about 2 cm from the pylorus (Fig. 1), with 2-0 absorbable suture. For patient 1 & 2, a complete 5–6 cm laparoscopic manual anastomosis (LMA) was performed using 2-0 absorbable suture in two planes. For patients 3 & 4, a standard stapled anastomosis (LSA) was performed creating 2 small holes, one in the gastric antrum and one in the bowel to introduce and shoot a 60 mm purple cartridge, the gastro-enterotomy was closed with 2-0 absorbable suture in one plane. Fig. 2 shows ileo-gastro anastomosis. A drain was left, and the incisions were closed and infiltrated with local anesthetics.

## 3 Results

Preoperative & postoperative outcomes are presented in Table 1, Table 2.

**Table 1:** Preoperative parameters.

Patient	1	2	3	4	Mean
**Demographics**					
Sex	F	F	M	F	3 (75.0 %)
Age	53	32	48	52	46.2 ± 9.7
Initial Weight (kg)	57.4	83.6	85.2	76.2	75.6 ± 12.7
Height (cm)	147	163	170	166	161.5 ± 10.0
Initial BMI (kg/m^2^)	26.5	31.4	29.4	27.6	28.7 ± 2.1
**Preop Labs**					
Fasting glucose (mg/dl)	194	254	117	337	225.5 ± 93.1
A1C (%)	9.5	15.2	8.4	10.1	10.8 ± 3.0
C peptide (ng/ml)	3.9	2.2	2.0	2.1	2.5 ± 0.9
Albumin (g/dl)	4.3	3.9	4.6	4.2	4.2 ± 0.2
Hemoglobin (g/dl)	13.6	12.8	15.6	14.5	14.1 ± 1.2
TGC (mg/dl)	176	220	149	201	186.5 ± 30.8
LDL (mg/dl)	112	106	57	151	106.5 ± 38.5
HDL (mg/dl)	40	39	54	57	47.5 ± 9.3
**Preop History**					
Time since diagnoses of T2D (years)	2	13	10	6	7.7 ± 4.7
Medications	MET 850 mg bid	MET 850 bid, INS 34 un qd	GLIM 5 mg qd, MET 1000 mg bid, LIR 1.2 mg per week, CAN 300 mg qd	MET 850 mg bid, LIN 5 mg qd	–
Comorbidities	HBP, HT	Diabetic neuropathy (previous finger amputation)	HBP, Subclinical HT,	HT, disk hernia, chronic knee pain	–
Previous Abdominal surgeries	No	CS, lap chole	No	Liposculpture	–
Smoking	No	No	No	No	–

BMI: body mass index; A1C: glycated hemoglobin; TGC: Triglycerides; LDL: Low–density lipoprotein; HDL: High–density lipoprotein; T2D: type–2 diabetes mellitus; GLIM: glimepiride; MET: metformin; INS: insulin; LIR: liraglutide; CAN: canagliflozin; LIN: linagliptin; HBP: high blood pressure; HT: hypothyroidism; CS: C–section; TAH: Total Abdominal Hysterectomy; Chole: Cholecystectomy.

**Table 2:** Postoperative parameters.

Patient	1	2	3	4	Mean
**Procedure**					
Anastomosis	LMA	LMA	LSA	LSA	–
Concomitant procedure	No	No	No	UHR	–
Surgical Time (min)	124	131	58	65	94.5 ± 38.3
Hospital Length of Stay (days)	2	2	2	2	2.0
Complications	No	No	No	No	–
Adverse effects	Biliary vomit(a)	Biliary vomit(a)	No	No	2(50 %)
**Follow-up**					
Follow-up (months)	18	18	17	6	14.7 ± 5.8
Weight (kg)	48.2	75.3	74.7	74.4	
BMI (kg/m^2^)	22.3	28.3	25.8	26.9	25.8 ± 2.5
%Excess Weight Loss	278	48.8	81.3	24.3	108.1 ± 115.6
BMI reduction (kg/m^2^)	4.2	3.1	3.6	0.7	2.9 ± 1.5
Fasting glucose (mg/dl)	84	140	98	130	113 ± 26.3
A1C (%)	5.5	8.1	5.9	7.3	6.7 ± 1.2
A1C (%) reduction	4.0	7.1	2.5	2.8	4.1 ± 2.1
Fasting glucose (mg/dl) reduction	93	114	7	207	105.2 ± 82.1
Albumin (g/dl)	3.1	4.1	4.8	3.5	3.8 ± 0.7
Hemoglobin	15.4	13.2	15.8	13.8	14.5 ± 1.4
TGC (mg/dl)	65	135	121	150	117.7 ± 37.1
LDL (mg/dl)	51	98	71	88	77 ± 20.6
HDL (mg/dl)	35	38	50	54	44.2 ± 9.1
TGC reduction	111	85	−28	6	43.5 ± 65.3
LDL reduction	61	8	−14	63	29.5 ± 38.5
HDL increase	21	−1	−4	−3	3.2 ± 11.8
Medications	No	MET 850 mg bid, GLIM 1cada 12	No	MET 850 mg qd	–
Improve T2D	Yes	Yes	Yes	Yes	–
Partial remission	No	No	No	No	–
Complete remission	Yes	No	Yes	No	–
Other comorbidities in complete remission	HBP	–	HBP	Chronic Knee pain	–

GIA–B: Gastroileal Anastomosis Bypass; LSA: Laparoscopic Stapled Anastomosis; LMA: Laparoscopic Manual Anastomosis; UHR: Umbilical Hernia Repair; BMI: Body Mass Index; A1C: Glycated hemoglobin; TGC: Triglycerides; LDL: Low–density lipoprotein; HDL: High–density lipoprotein; GLIM: glimepiride; MET: metformin; GLIM: glimepiride; T2D: type–2 diabetes mellitus; HBP: High Blood Pressure. (a) Patient 4 started with biliary vomit 5 months after surgery every day for 7 days. An endoscopy was performed with report of erosive gastritis and biliary content in stomach, no esophagitis. Biliary vomit was controlled with conservative treatment. Patient 5 started with biliary vomit 1 month after the surgery that persisted for 3 months 1 per week. Biliary vomit was controlled with conservative treatment.

## 4 Discussion

Several lines of evidence and logic justify contemplating the use of bariatric/metabolic operations in lower-BMI patients with T2D that are not adequately controlled with behavioral/pharmaceutical interventions [5]. BMI remains being considered as an eligibility criterion despite this proof. The American Diabetes Association (ADA) [6] and other international diabetes organizations proposed a BMI threshold of 30 kg/m^2^ (27.5 in Asian patients) for considering metabolic surgery in patients with T2D with uncontrolled hyperglycemia. During last decade the popularity of SG has growth, being today the metabolic and bariatric procedure that is performed most frequently around the world [7], and no necessarily the most effective. This inclination enables us to realize the importance not just of effectiveness, but also of safety, ease and price when choosing physicians or patients for a bariatric or metabolic operation. Even with this broad range of metabolic procedures, only a tiny proportion of qualified individuals may have access to operation. Under those circumstances, we think is mandatory to continue exploring for new techniques that provides a balance among effectivity, safeness, efficiency, simplicity and cost.

### 4.1 Technical considerations

GIA-B is a simplification of SASI-B popularized by Mahdy in 2016 where an anastomosis between the ileum (300 cm from the ileocecal valve) and the stomach is performed, similar to SASI-B, but without a prior SG. GIA-B's physiological mechanism is like many other metabolic processes, the rapid delivery of nutrients to the lower intestine increases stimulation of L-cells (“lower intestinal hypothesis”), which results in increased secretion of hormones that enhance insulin release and/or insulin action (for example, GLP-1), and a subsequent decrease in blood glucose levels [8,9]. In GIA B, there is a distinction with other type of bypasses, where the stomach is splitted or removed. In GI A-B, the stomach is intact and only the antrum will be altered to produce anastomosis. Other distinction is that, in GI A-B, there is a significant reduction in the transit of food through the first part of the bowel, but not a complete exclusion of the duodenum and jejunum. It can be hypothesized that the lack of exclusion of the first part of the bowel can have less impact on the glucose reduction but decreases the possibility of nutritional deficiencies.

### 4.2 Values A1C, remission and medications

It is remarkable the metabolic effect seen in our cases, with a mean follow-up of 14.7 months we found an important mean reduction in A1C (4.1 %) and mean fasting glucose (105 mg/dl). Also, the average postoperative fasting glucose (113 mg/dl) was similar to those reported for other more complex metabolic procedures [[10], [11], [12]]. Complete remission of T2D was found in 50 % [2] of the cases while the remaining did not reach complete remission but improved considerably their parameters.

When we assess metabolic parameters, we note that in each situation A1C and the demands for medicines decreased, supporting an intrinsic metabolic pathway. We believe that this effect is produced by a strong physiological mechanism when the first part of the bowel is bypassed and non-well digested food reached distal ileum leading to production of GIP, GLP-1 and PYY, as occurs in most common metabolic procedures.

### 4.3 Weight loss

In this study GIA-B reached a mild weight loss, with BMI decreased –2.9 kg/m^2^, similar to the meta-analysis performed by Rubio-Almanza et al. [13].

### 4.4 Lipid profile

All the patients in this sequence had preoperative hypertriglyceridemia and all of them had lipid changes. As expected, the weight loss and the metabolic effect induced by the surgeries contributed to improve the lipid profile, especially triglycerides, which improved in all of them at the final follow-up.

### 4.5 Complications & adverse effects

No intra-or post-operative complications were found, likely due to the few cases recorded. While this document does not demonstrate that GIA-B may have lower levels of complication than other metabolic processes, the nature and ease of the procedure make logical to believe that these surgeries could reduce the complication rate. Of the 4 patients who received GI A-B, 2 (50 %) had biliary vomit that was conservatively handled and did not require surgical treatment until this document was written. The physiological mechanism that provoke this adverse effect is well understood, the arrival of biliary content to the stomach. In view of the low rate of biliary vomit seen in other procedures, such as SASI-B or OAGB/MGB [14,15], we think that 50 % of patients with biliary vomit is considered elevated. The cause of it is beyond our understanding, although we think that a greater number of cases will reduce the rate of this adverse effect.

In order to open research into this new process, we introduce this preliminary research, which simplify other already defined metabolic methods. We think that the principal limitation of this research is the scarce number of patients and the lack of serum levels of pre and post-operative hormones, specially GLP-1 and PYY. We conclude that GIA-B have a metabolic impact reaching improvement in homeostatic parameters in all patients assessed, but a longer follow-up and more patients are necessary for a better evaluation of its metabolic effects. It is probably that the metabolic effect that we found in this series of cases can be magnified in patients with less time between the diagnoses of T2D and surgery or those with better glycemic control prior the surgery. GIA-B appears to be technically easier than other metabolic techniques and the cost is considerably lower especially for the saving of cartridges. We believe that there are room for easy and inexpensive methods in particular for T2D patients with low body mass index. However, more studies are needed to validate this technique.

## Sources of funding

None.

## Ethical approval

This study was performed prior the authorization of the ethical committee of the Boca del Rio General Hospital at Veracruz, Mexico registered with number 018/2016.

## Consent

Written informed consent was obtained from the patients for publication of this case report and accompanying images. A copy of the written consent is available for review by the Editor-in-Chief of this journal on request.

## Author contribution

The authors Raquel Colorado-Subizar and Rey J. Romero have contributed to study concept and design, data collection, data analysis and interpretation and writing the paper.

## Registration of research studies

researchregistry5220.

## Guarantor

Rey Jesus Romero.

## Provenance and peer review

Not commissioned, externally peer-reviewed.

## CRediT authorship contribution statement

**Rey Jesús Romero:** Conceptualization, Methodology, Validation, Formal analysis, Investigation, Resources, Data curation, Writing - review & editing, Visualization, Supervision. **Raquel Colorado-Subizar:** Methodology, Software, Validation, Formal analysis, Investigation, Writing - original draft, Visualization.

## Declaration of Competing Interest

The authors Raquel Colorado-Subizar and Rey Jesus Romero declare no conflict of interest.
